# Scimitar syndrome: a rare disease

**DOI:** 10.31744/einstein_journal/2021AI6683

**Published:** 2021-12-14

**Authors:** Bruna Provenci, Roberta Karla Barbosa de Sales, Eduardo Kaiser Ururahy Nunes Fonseca, Rodrigo Caruso Chate

**Affiliations:** 1 Universidade de São Paulo Faculdade de Medicina Hospital das Clínicas São Paulo SP Brazil Instituto do Coração, Hospital das Clínicas, Faculdade de Medicina, Universidade de São Paulo, São Paulo, SP, Brazil.; 2 Universidade de São Paulo Faculdade de Medicina Hospital das Clínicas São Paulo SP Brazil Hospital das Clínicas, Faculdade de Medicina, Universidade de São Paulo, São Paulo, SP, Brazil.

**Figure 1:**
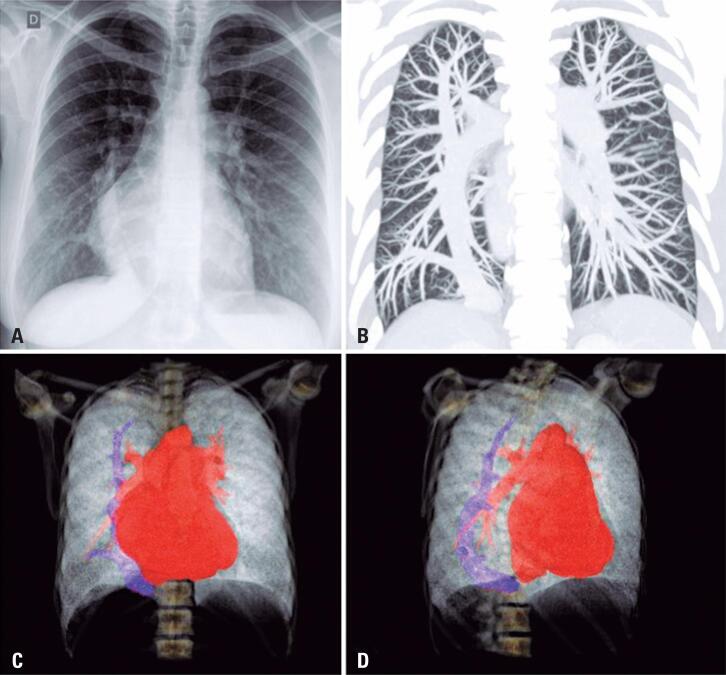
Chest images. (A) Posteroanterior chest radiography demonstrating a calibrous and slightly arched tubular structure, with a pathway relatively parallel to the right cardiac contour, towards the cavoatrial junction, associated with signs of right atrial enlargement; (B) Tomographic image with maximum intensity projection, coronal reformatting, showing a large caliber anomalous vein with a slightly arched pathway in the right lung, draining into the region of the cavoatrial junction; (C and D) Three-dimensional reformatting of chest computed tomography with intravenous iodinated contrast agent, anterior (C) and right anterior oblique (D) projections, showing a large-caliber anomalous vein with an arched path along the craniocaudal axis of the right lung, draining into the cavoatrial junction, compatible with scimitar

A 34-year-old patient with a history of asthma, taking inhaled corticosteroids and bronchodilators, presented with worsening dyspnea (functional class III) and reports of syncope. On physical examination, pulmonary auscultation was normal with pulse oximetry of 96% in room air. On cardiac auscultation, the rhythm was regular with fixed splitting S2 and systolic murmur 2+/6+ on left sternal border. On abdominal examination, the liver was palpable 2cm below the right costal border. There were no other signs of heart failure. Chest radiography was performed during the investigation, which showed a calibrous tubular structure slightly arched towards the right atrium and part of the cardiac area occupying the right hemithorax ( Figure 1A ). Chest CT scan revealed a large caliber anomalous vein in the right lung, draining into the cavoatrial junction ( Figure 1B ). Three-dimensional computed tomography scan using intravenous contrast showed a thick anomalous vein arched along the craniocaudal axis of the right lung, draining into the cavoatrial junction, compatible with scimitar ( Figures 1C and 1D ).

The patient underwent right and left cardiac catheterization, which revealed an *ostium secundum* atrial septal defect (ASD) of 18mm, left-to-right flow with repercussions in the right chambers, and anomalous drainage of the right pulmonary veins into the inferior vena cava. A fine caliber arterial branch of the celiac trunk irrigated a small portion of the lower lobe of the right lung. Pulmonary hyperflow was shown with pulmonary artery pressure measurements of 30x15 and mean of 21, adequate pulmonary vascular resistance (PVR) of 0.95W, with pulmonary capillary wedge pressure of 10, and right atrial pressure of 8. Qp/Qs (pulmonary/systemic flow) was 1.74, indicating left-right shunt.

Scimitar syndrome is a rare disorder characterized by anomalous venous drainage from the lung directly into the inferior vena cava. The nomenclature of the syndrome comes from the similarity of the shape of the image to a curved Turkish sword or scimitar ( Figure 1A ). The disease may be associated with right lung hypoplasia, bronchial tree abnormalities, dextrocardia, cardiac abnormalities, and systemic arterial supply to the right lung from the aorta or bronchial arteries.^( 1 )^

The patient in the case has an atrial septal defect, the most common cardiac abnormality (65%), described by a multicenter study that included 68 patients with scimitar who underwent surgical approach.^( 2 )^

The symptoms are varied and most often occur due to cardiac malformations, with the diagnosis usually being made in childhood (infantile form). Patients who present with the infantile form tend to have a more severe disease with a worse prognosis, associated with pulmonary hypertension. Diagnosis in adulthood (adult form) is less common, and patients usually have milder symptoms or are asymptomatic.^( 3 )^

Surgical intervention can be a safe and effective treatment for selected cases, and is most often performed in childhood. Adult-age interventions may also be indicated, usually in symptomatic individuals.^( 4 )^ The type of intervention depends on the associated anomalies, and the treatment basically consists of redirecting the venous return to the left atrium. The approaches can be performed by surgery or by endovascular methods that aim to occlude the aorta-pulmonary collateral circulation.^( 5 )^
