# Supplementary material for: Scimitar syndrome: a rare disease
Source: Einstein (Sao Paulo). 2021 Dec 14;19:eAI6683. doi: 10.31744/einstein_journal/2021AI6683 (PMC8687649; doi:10.31744/einstein_journal/2021AI6683)
Supplement: Translation

# Translation: Síndrome de cimitarra: uma doença rara

**Authors:** Bruna Provenci, Roberta Karla Barbosa de Sales, Eduardo Kaiser Ururahy Nunes Fonseca, Rodrigo Caruso Chate

**Affiliations:** 1 São Paulo SP Brasil Instituto do Coração, Hospital das Clínicas, Faculdade de Medicina, Universidade de São Paulo, São Paulo, SP, Brasil.; 2 São Paulo SP Brasil Hospital das Clínicas, Faculdade de Medicina, Universidade de São Paulo, São Paulo, SP, Brasil.

Paciente de 34 anos com histórico de asma, em uso de corticoide inalatório e broncodilatador, apresentava piora de dispneia (classe funcional III) e relatos de síncope. Ao exame físico, a ausculta pulmonar era normal com oximetria de pulso de 96% em ar ambiente. Na ausculta cardíaca, o ritmo era regular, com B2 desdobrada fixa e sopro sistólico 2+/6+ em borda esternal esquerda. Ao exame abdominal, o fígado era palpável 2cm abaixo do rebordo costal direito. Não havia outros sinais de insuficiência cardíaca. Foi realizada radiografia de tórax durante a investigação, que evidenciou estrutura tubular calibrosa levemente arqueada em direção ao átrio direito e parte da área cardíaca ocupando o hemitórax direito ( Figura 1A ). Tomografia de tórax revelou veia anômala de grosso calibre no pulmão direito, drenando na junção cavoatrial ( Figura 1B ). A tomografia tridimensional utilizando contraste intravenoso mostrou veia anômala espessa arqueada ao longo do eixo craniocaudal do pulmão direito, drenando na junção cavoatrial, compatível com cimitarra ( Figuras 1C e 1D ).

**Figura 1:** Imagens do tórax. (A) Radiografia de tórax em posteroaterior demonstrando estrutura tubular calibrosa e levemente arqueada, com trajeto relativamente paralelo ao contorno cardíaco direito, dirigindo-se à junção cavoatrial, associada a sinais de aumento do átrio direito; (B) Imagem tomográfica com projeção de intensidade máxima, reformatação coronal, mostrando veia anômala de grosso calibre com trajeto levemente arqueado no pulmão direito, drenando na região da junção cavoatrial; (C e D) Reformatações tridimensionais de tomografia de tórax com utilização de contraste iodado endovenoso, projeções anterior (C) e oblíqua anterior direita (D), revelando veia anômala de grosso calibre com trajeto arqueado ao longo do eixo craniocaudal do pulmão direito, drenando na junção cavoatrial, compatível com cimitarra

A paciente foi submetida a cateterismo cardíaco direito e esquerdo, que evidenciou comunicação interatrial (CIA) do tipo *ostium secundum* 18mm, fluxo esquerda-direita com repercussão em câmaras direitas e drenagem anômala de veias pulmonares direitas para veia cava inferior. Um ramo arterial do tronco celíaco de fino calibre irrigava pequena porção do lobo inferior do pulmão direito. Foi evidenciado hiperfluxo pulmonar com medidas de pressão de artéria pulmonar de 30x15 e média de 21, resistência vascular pulmonar (RVP) adequada (0,95W) com capilar pulmonar de 10 e pressão em átrio direito de 8. O Qp/Qs (fluxo pulmonar/sistêmico) era de 1,74, indicando o *shunt* esquerda-direita.

A síndrome de cimitarra é uma doença rara caracterizada pela drenagem venosa anômala do pulmão direto para veia cava inferior. A nomenclatura da síndrome vem da semelhança do formato da imagem a uma espada turca curva ou cimitarra ( Figura 1A ). A doença pode estar associada a hipoplasia pulmonar direita, anormalidades na árvore brônquica, dextrocardia, anormalidades cardíacas e suprimento arterial sistêmico ao pulmão direito originário da aorta ou artérias brônquicas.^( 1 )^

A paciente do caso apresenta defeito no septo interatrial, sendo a anormalidade cardíaca mais comum (65%) descrita por um estudo multicêntrico que incluiu 68 pacientes com cimitarra submetidos à abordagem cirúrgica.^( 2 )^

Os sintomas são variados e, na maioria das vezes, ocorrem devido às malformações cardíacas, com o diagnóstico sendo realizado geralmente na infância (forma infantil). Os pacientes que apresentam a forma infantil tendem a apresentar doença mais grave e de pior prognóstico, associada à hipertensão pulmonar. O diagnóstico na vida adulta (forma do adulto) é menos comum, e, geralmente, os pacientes apresentam sintomas mais leves ou são assintomáticos.^( 3 )^

A intervenção cirúrgica pode ser um tratamento seguro e eficaz para os casos selecionados e, na maior parte das vezes, é realizada ainda na infância. As intervenções na idade adulta também podem ser indicadas geralmente em indivíduos sintomáticos.^( 4 )^O tipo de intervenção depende das anomalias associadas, sendo que o tratamento basicamente consiste em redirecionar o retorno venoso para o átrio esquerdo. As abordagens podem ser realizadas por meio de cirurgia ou por métodos endovasculares que visam à oclusão da circulação colateral aortopulmonar.^( 5 )^
